# The PRogram In Support of Moms (PRISM): study protocol for a cluster randomized controlled trial of two active interventions addressing perinatal depression in obstetric settings

**DOI:** 10.1186/s12884-019-2387-3

**Published:** 2019-07-22

**Authors:** Tiffany A. Moore Simas, Linda Brenckle, Padma Sankaran, Grace A. Masters, Sharina Person, Linda Weinreb, Jean Y. Ko, Cheryl L. Robbins, Jeroan Allison, Nancy Byatt

**Affiliations:** 1 University of Massachusetts Medical School, 55 Lake Avenue, Worcester, MA 01655 USA; 2 Department of Obstetrics and Gynecology, University of Massachusetts Medical School, 119 Belmont Street, Worcester, MA 01605 USA; 3 Department of Pediatrics, University of Massachusetts Medical School, 55 Lake Avenue, Worcester, MA 01655 USA; 4 Department of Psychiatry, University of Massachusetts Medical School, 55 Lake Avenue, Worcester, MA 01655 USA; 5 Department of Quantitative Health Sciences, University of Massachusetts Medical School, 55 Lake Avenue, Worcester, MA 01655 USA; 6 Department of Family Medicine and Community Health, University of Massachusetts Medical School, 55 Lake Avenue, Worcester, MA 01655 USA; 7 Department of Obstetrics and Gynecology, UMass Memorial Health Care, 119 Belmont Street, Worcester, MA 01605 USA; 8 Department of Psychiatry, UMass Memorial Health Care, 6 Lake Avenue, Worcester, MA 01655 USA; 9 Fallon Health, Worcester, MA USA; 10 Centers for Disease Control and Prevention, Atlanta, GA USA; 11 U.S. Public Health Service, Comissioned Corps, Maryland, USA

**Keywords:** Pregnancy, Postpartum, Perinatal, Depression, Integrated care, Methods, Protocol, Intervention, Implementation randomized controlled trial

## Abstract

**Background:**

Perinatal depression, the most common pregnancy complication, is associated with negative maternal-offspring outcomes. Despite existence of effective treatments, it is under-recognized and under-treated. Professional organizations recommend universal screening, yet multi-level barriers exist to ensuring effective diagnosis, treatment, and follow-up. Integrating mental health and obstetric care holds significant promise for addressing perinatal depression. The overall study goal is to compare the effectiveness of two active interventions: (1) the Massachusetts Child Psychiatry Access Program (MCPAP) for Moms, a state-wide, population-based program, and (2) the PRogram In Support of Moms (PRISM) which includes MCPAP for Moms plus a proactive, multifaceted, practice-level intervention with intensive implementation support.

**Methods:**

This study is conducted in two phases: (1) a run-in phase which has been completed and involved practice and patient participant recruitment to demonstrate feasibility for the second phase, and (2) a cluster randomized controlled trial (RCT), which is ongoing, and will compare two active interventions 1:1 with ten Ob/Gyn practices as the unit of randomization. In phase 1, rates of depressive symptoms and other demographic and clinical features among patients were examined to inform practice randomization. Patient participants to be recruited in phase 2 will be followed longitudinally until 13 months postpartum; they will have 3–5 total study visits depending on whether their initial recruitment and interview was at 4–24 or 32–40 weeks gestation, or 1–3 months postpartum. Sampling throughout pregnancy and postpartum will ensure participants with different depressive symptom onset times. Differences in depression symptomatology and treatment participation will be compared between patient participants by intervention arm.

**Discussion:**

This manuscript describes the full two-phase study protocol. The study design is innovative because it combines effectiveness with implementation research designs and integrates critical components of participatory action research. Our approach assesses the feasibility, acceptance, efficacy, and sustainability of integrating a stepped-care approach to perinatal depression care into ambulatory obstetric settings; an approach that is flexible and can be tailored and adapted to fit unique workflows of real-world practices.

**Trial registration:**

ClinicalTrials.gov Identifier: NCT02760004, registered prospectively on May 3, 2016.

**Electronic supplementary material:**

The online version of this article (10.1186/s12884-019-2387-3) contains supplementary material, which is available to authorized users.

## Background

Perinatal depression (Major and Minor Depressive Disorder occurring during pregnancy or within one year of delivery), affecting upwards of one in seven women, is the most common complication of pregnancy [1]. Despite being associated with negative maternal [2], birth [3], infant [4] and child outcomes [5–7], which are mitigated by effective treatment [8] that includes psychopharmacology and psychotherapy [9], perinatal depression remains under-diagnosed [10–13] and under-treated [14]. Recognizing the magnitude of this problem, professional societies, policy makers, and other stakeholders recommend universal screening of pregnant and/or postpartum women using validated tools [1, 15–18].

Screening for depression is widely accepted by perinatal women and Ob/Gyn providers [19, 20]. However, screening alone does not translate into treatment participation given multi-level barriers to care. Provider and systems-level barriers include: (a) lack of obstetric provider training in technical aspects of depression care [20–22] and relevant communication skills [23]; (b) absence of standardized processes and procedures for integrateding obstetric and depression care [23, 24]; (c) lack of mental health providers willing to treat pregnant women [24]; (d) lack of referral networks [23–27]; and, (e) inadequate capacity and resources to ensure depression evaluation, treatment, follow-up, and care coordination [23–29].

The obstetric practice setting holds significant promise as a place to address depression in perinatal women, with approximately six million pregnancies occurring annually in women age 15–44 years in the United States [30]. Obstetric providers are often the only medical professionals that young women see and thus their practices need supports to adequately address depression in perinatal women [20]. Even in low risk pregnancies, women will have 12–15 visits in under a year [31], thus providing ample opportunities to screen in the context of obstetric care. In an effort to set relevant standards, the Council on Patient Safety in Women’s Health Care developed an evidence informed patient safety bundle [15]. The bundle provides broad direction and is well-aligned with the American College of Obstetricians and Gynecologists’ recommendations to screen in the context of systems that ensure effective diagnosis, treatment, and follow-up [1, 15].

The care of women is lagging-behind changing care standards [1, 15] and will be further challenged to advance without purposeful efforts aimed at innovating mental health care delivery in obstetric settings. Translating integrated, stepped-care models, as in models that provide specific treatment protocols in response to illness severity [32], to obstetric settings could provide a solution. It is well-established that these models effectively integrate depression treatment into primary care settings and improve the quality of mental health care and depression outcomes [33]. Such approaches have been introduced and evaluated in outpatient obstetric practices with evidence supporting feasibility, effectiveness, and acceptability [34]. However, it remains an emerging field with current data focused mostly on process measures [34] and with few studies assessing symptom and outcome improvements for women and children [35–39].

Our team developed and implemented a first-in nation, state-wide, population-based program, the Massachusetts Child Psychiatry Access Program for Moms (MCPAP for Moms), designed to enhance the capacity of obstetric providers to address perinatal depression [40–42]. In so doing, we discovered opportunities to address additional gaps in care. To achieve full remission of depression symptoms, women with perinatal depression must: 1) be recognized via screening; 2) be assessed; 3) initiate treatment; 4) receive adequate treatment; and, 5) respond to treatment [43, 44]. MCPAP for Moms provides access to resources, however it does not include the more intensive implementation and follow-up components that Ob/Gyn practices and their patients need [45]. Thus, building on MCPAP for Moms and integrated care models, we have designed and piloted [46] a more comprehensive practice-level intervention, the PRogram In Support of Moms (PRISM) [35].

The aim of this paper is to describe the methods of a large-scale, multi-site cluster-randomized controlled trial (RCT) study protocol comparing two active interventions in addressing perinatal depression in obstetric settings. The first intervention is a population-based health program recognized as enhanced usual care (MCPAP for Moms) that provides all Massachusetts obstetric practices with access to telephonic psychiatric consultation and mental health resources through care coordination services [40, 42]. The second intervention is our practice-level intervention (PRISM) [35, 47], a proactive, multifaceted, and practical intervention that pairs MCPAP for Moms with intensive implementation support and follow-up, helping obstetric practices implement and sustain depression care, thus ensuring that women complete the entire depression care pathway [44, 48].

## Methods/design

### Study Design & Overview

The first study phase (phase 1 or the run-in phase), which has been completed, involved recruitment of practice and patient participants as a run-in to demonstrate feasibility for participation in the second phase of the RCT (see Additional file 1 for study timeline). To inform randomization, the prevalence of depressive symptomatology, clinical characteristics, and treatment participation were examined among phase 1 patient participants that received care in the ten participating practices (see Additional file 2 for study centers). The second phase (phase 2), which is ongoing and not yet completed, compares the two active interventions in a 1:1 cluster RCT in which 10 Ob/Gyn practices are randomized to MCPAP for Moms or PRISM (MCPAP for Moms plus practice-level intervention). The randomization approach was informed by our prior pilot work [35, 47] and was done at the practice level because: [1] practices are the natural intervention unit thus avoiding “contamination” when exposed and unexposed physicians are in the same office [49], and [2] patient-level randomization is less feasible in this type of intervention [49]. All patient participants are being recruited by the study team outside of the practice environment to decrease biases introduced by patient participants potentially receiving more attention or intervention in comparison to patients in the same practice but not enrolled in the study. Patient participants enter the designated study arm based on where they receive care; they are blinded to whether care is received at a practice randomized to MCPAP for Moms or PRISM. Patient participants can be recruited at any point during pregnancy through 3 months postpartum to ensure patient participants with differential depressive symptom onset times. Patient participants to be recruited in phase 2, depending on their recruitment and first interview time point (4–24 weeks gestation, 32–40 weeks gestation, or 1–3 months postpartum), will be followed 2–4 more times until 12 months postpartum.

Overall this study aims to compare the effectiveness of MCPAP for Moms alone versus PRISM in improving depressive symptom severity (primary outcome) and treatment participation in pregnancy through 12 months postpartum (secondary outcome). We hypothesize that perinatal patient participants receiving care from practices enrolled in PRISM will experience more improvement in depression symptoms and increased treatment participation, than patient participants receiving care in practices with MCPAP for Moms only. Secondarily, we will examine provider and staff fidelity to PRISM, and estimate costs and indicators of potential savings of PRISM.

The University of Massachusetts Medical School Institutional Review Board (UMass IRB) human subjects committee approved this study, providing review for the two UMass Memorial Health Care-affiliated practices and one practice without an IRB. In addition, six IRBs, affiliated with the seven other participating practices, reviewed the study. Of these, four ceded review to UMass IRB and two retained independent review and approval. The CDC was not engaged in conducting the research so its IRB approval was not needed. The ClinialTrials.gov registry number is NCT02760004. The study started in October 2015. As of July 2019, all practice recruitment, patient participant recruitment for the run-in phase, and randomization is complete. The phase 2 RCT patient participant recruitment and data collection is active and on-going. It is anticipated that patient participants’ postpartum follow-up and thus data collection will be complete in 2021. Data cleaning and primary planned analyses are planned to be completed in 2022 (Additional file 1).

### Phase 1: Run-in phase – feasibility of practice and patient recruitment and data collection

Study phase 1 focused on practice recruitment and characterization; this was designed to inform phase 2 randomization with covariate adaptive matching [50–52]. Critical to practices’ randomization was a demonstrated ability to carry out study procedures including processes that allowed patient participant recruitment by the study team via phone, outside of the practice setting. To meet criteria for randomization, each practice needed to have at least 25 patient participants enrolled and at least one patient participant who screened positive for depression.

### Practice recruitment, including provider and staff participants

Practices were recruited from July 2015 to February 2017 with the goal of geographic distribution across the state, diversity of practice environment (e.g. academic, private practice), and socioeconomic and racial diversity among the patients they serve. Within practices, participating providers and staff included attending and resident physicians, advanced practice nurses, nurses, midwives, patient care assistants, social workers, administrative support staff, and others serving the practices’ patients.

### Practice-level measurements, assessments, data collection procedures

Each practice completed a multi-component Practice Readiness to Evaluate and address Perinatal Depression scale (PREPD), to be described in a forthcoming manuscript. This produced a cumulative baseline Practice Readiness Index (PRI) score that indicated the extent to which individual practices had integrated depression care, and was used to inform randomization and provided a benchmark against which to measure future change.

Provider participants completed a modified Smoking Knowledge, Attitudes, and Practices Instrument (S-KAP) [53], a 46 item tool that we adapted for use in obstetric settings. To capture data not included in the measures, we added questions with a five-level Likert Scale inquiring about provider participant perspectives of the intervention components’ usefulness, and their perceptions of their own self-efficacy to manage depression, adequate access to depression care, ability to ensure patients get needed treatment in a timely manner, and ability to meet the mental health needs of women.

### Patient participant eligibility/ineligibility criteria and recruitment time points

Patient participant enrollment eligibility for the run-in phase was less stringent than in phase 2 (see *Phase 2* below). For example, patients were eligible for recruitment regardless of depressive symptomatology based on the Edinburgh Postnatal Depression Scale (EPDS). Additionally, patients were not excluded from recruitment based on a positive 4Ps (Pregnancy, Past, Partner, Parents) substance use disorder screen or Mood Disorder Questionnaire (MDQ) bipolar disorder screen. To include patient participants with differential depressive symptom onset times, patients were recruited during pregnancy and through three months postpartum and had only a single point of study contact. The broad inclusion criteria in the run-in allowed us to examine the prevalence of depressive symptoms and other conditions and demographics within a broader sample from the practices. This data was then used to inform randomization and to phase 2 recruitment.

### Phase 2: Randomized controlled trial

Study phase 2, which is ongoing, started with practice randomization into the two intervention arms: [1] MCPAP for Moms only, and [2] PRISM (see *Intervention descriptions* below). Randomization was informed by practice-level and patient-level data collected in phase 1. Patient participant recruitment procedures are similar to those in phase 1: they are recruited by study staff via phone outside of the practice in a blinded fashion. However, in phase 2, patient participants are enrolled for longitudinal data collection and serial assessments, and are to be maintained in the study until 12 months postpartum.

#### Randomization and allocation procedures

Although cluster randomization provides protection against heterogeneity that may occur due to patient participants in a practice not being independent, serious imbalances can occur as practices often have vastly different patient profiles; thus, the baseline assumption that the treatment groups are equal, subsequently hindering any causal inferences, is threatened. To protect against this threat, a restricted randomization technique using minimization was employed allowing for balanced allocation of practices to treatment arms on important patient characteristics. Data on practice readiness, quantified as the Practice Readiness Index (PRI) score, patient depressive symptom prevalence and severity as per the EPDS, as well as socioeconomic status indicated by insurance status, and non-white race/ethnicity, gathered during study phase 1, were used to characterize practices and formed the basis for restricted randomization. Specifically, covariate adaptive matching using Mahalanobis matching randomization was used [50, 51]. Mahalanobis distances were used to determine the optimal matches of practices, given the covariates under consideration; then, practices were randomized within the matched pairs. Five practices were randomized to each of the two interventions being evaluated.

Table 1 displays the results of the randomization process. The randomization procedure matched practices on five factors deemed most relevant a priori; distributions of these factors are not statistically significally different between the two intervention groups.

**Table 1:** Results of the factors used in matching during Randomization and presented by intervention arm

Factor	Mean (SD)		Difference	*P*-value
	MCPAP for Moms (*n* = 5 practices)	PRISM (n = 5 practices)		
EPDS Score*	14.19 (0.91)	14.92 (2.31)	−0.74	0.53
Prevalence Depression	23.1 (6.66)	20.01 (5.92)	3.08	0.61
Prevalence Public Insurance	48.76 (32.16)	57.95 (36.4)	−9.18	0.68
Prevalence Non-White	36.38 (16.95)	42.51 (23.75)	−6.14	0.69
Practice Readiness Index^≠^	7.07 (1.99)	8.27 (0.82)	−1.20	0.25

Table 1 represents the results of the reweighted Mahalanbois Distance/Covariate Adaptive matching procedure. Matching was done on the five factors deemed most relevant a priori to matching procedures. Factors are reported as mean (standard deviation) by intervention arms in addition to differences and associated *p*-values. Intervention arms were the PRogram In Support of Moms (PRISM) versus Massachusetts Child Psychiatry Access Program (MCPAP) for Moms interventions. *Mean Edinburgh Postnatal Depression Scale (EPDS) score among those with an EPDS ≥10. ^≠^ The Practice Readiness Index (PRI) was a cumulative score (total possible 16 points) derived from the multi-component Practice Readiness to Evaluate and address Perinatal Depression (PREPD) scale.

#### Interventions

Both interventions have been described elsewhere [35, 40, 42, 47], including a table of key characteristics [35], and thus are briefly outlined here.

##### MCPAP for Moms alone [35, 40, 42]

MCPAP for Moms is a population-based, program available to all Ob/Gyn practices throughout the state of Massachusetts. MCPAP for Moms consists of training and toolkits, provider access to perinatal psychiatric consultation via telephone and face-to-face as needed, and facilitation of access to mental health resources and referrals cataloged in a living inventory that is regularly updated. Statewide provider access to MCPAP for Moms is essential to maintain ethical standards.

##### PRogram in support of Moms (PRISM) [35, 47]

PRISM includes MCPAP for Moms plus additional training, technical assistance, and implementation and change management support, utilizing the ten step model of Addressing Problems Through Organizational Change [35, 54, 55]. PRISM is a multi-pronged intervention that has core and optional elements. PRISM aims for practices to engage in systematic depression screening, assessment, and stepped treatment response; additionally, it provides proactive treatment engagement and patient monitoring of all patients with depressive symptoms to ensure that patients initiate, receive, and respond to treatment. PRISM is customizable to each practice; in fact, it evolves over time as practices iteratively identify steps to achieve their self-identified goals. Core elements include: 1) an assessment of practice readiness to implement PRISM (the PREPD evaluation); 2) identification of practice-level champion(s) and a work group to implement change plans through practice-identified strategies and tactics; 3) provider and staff training and toolkit customization to facilitate stepped-care within the specific practice environment; 4) on-site implementation assistance by the investigative team; and, 5) ongoing sustainment meetings and support to sustain change. As part of the proactive patient monitoring, a non-physician navigator maintains a registry of all patients with a positive depression screen. The navigator facilitates treatment engagement through motivational interviewing and provision of psychoeducation, while performing direct patient outreach, offering additional referrals, and ensuring patient engagement (and re-engagement as needed) in recommended medication treatment and attendance at mental health appointments via weekly to at least monthly follow-up calls. Patient monitoring also includes ongoing observation of symptoms thus allowing providers to determine whether a higher level of stepped-care is warranted at any stage in the treatment process. Finally, a MCPAP for Moms perinatal psychiatrist reviews cases twice per month with the navigator to provide expert consultation and education on management and treatment engagement.

#### Practice-level assessments

Practices that successfully completed phase 1 were randomized as indicated above. Their phase 1 data constitutes a baseline, with subsequent data to be collected yearly and at study completion. Table 2 details practice participation data, adherence, fidelity, and acceptability data to be collected longitudinally.

**Table 2:** Practice and Provider/Staff Participant-Level Measures

Outcome/ Endpoint	Measure	Administration	Time Points*		
			Baseline	Mid-point	Final
Practice Profile	Practice characteristics	Self-administered	√		
Participation	Training log	Research Coordinator (RC) collects	ongoing		
	Assessment of Practice Readiness to Evaluate and address Perinatal Depression (PREPD)	RC collects and self-administered components	√	√	√
	Five-level Likert scale for obstetric providers/staff	Self-administered online or paper	√	√	√
Adherence And Fidelity	Assessment of Practice Readiness to Evaluate and address Perinatal Depression (PREPD)	RC collects and self-administered components	√	√	√
	Measures of fidelity	Self-administered online	Every 3 months for PRISM practices following intervention implementation		
Knowledge, Attitudes, Acceptability, And Practices	Five-level Likert scale for obstetric providers/staff	Self-administered online or on paper	√	√	√

Table 2 represents the measures and time points at which practice participation, adherence, fidelity, and acceptability data are to be collected according to the PRogram In Support of Moms (PRISM) protocol. Baseline data was collected as part of the phase 1 run-in data. *Time points refer to stage of PRISM study, with baseline being prior to the start of patient recruitment, mid-point being one year after intervention implementation and final being two years after implementation.

#### Patient participant recruitment procedures and eligibility/ineligibility criteria

The study team worked with the practices to implement protocols for systematically identifying patient participants. Lists of potential participants were compiled by the offices and shared securely with the study team after appropriate permissions were secured and safeguards were in place. Some practices used an opt-in approach where the office staff collected lists of patients agreeing to be contacted by the study staff and forwarded that list securely to the study staff. Other practices securely provided comprehensive lists of practice patients to study staff and thus the study team was the first point of contact.

Study flyers, fact sheets, HIPAA authorization forms, and introductory letters signed by each respective practice director were mailed to patients asking them to opt out if uninterested. Approximately two weeks after the mailing, script-based telephone recruitment efforts ensued. During phone recruitment, potential patient participants verbally consented to study participation and gave authorization for access to medical records.

Phone recruitment scripts included the EPDS, the 4Ps, and the MDQ. The EPDS is a validated, 10-item screening questionnaire that is widely used to assess depression during pregnancy and the postpartum period [44]. The intensity of depressive symptoms is rated for the preceding seven days, and each item is scored on a 4-point scale for a total score range of 0–30, with higher scores reflecting a greater severity of symptoms [44]. The 4Ps is a validated screen [56] used to look for evidence of active substance abuse and the MDQ [57] is used to screen for bipolar disorder. Finally, other factors associated with mental health treatment participation as well as education, insurance status, and race/ethnicity are collected during phone recruitment.

Patient participants are considered eligible for phase 2 only if they demonstrate depressive symptomatology on the EPDS, with a score ≥ 10. Patient participants are ineligible for participation when there is evidence of active substance abuse on the 4Ps, or a positive screen for bipolar disorder via the MDQ. This is in contrast to phase 1, where the EPDS, MDQ, and 4Ps measures were used only to estimate rates of positives screens of depression, bipolar disorder, and active substance use and give projections for phase 2 recruitment, rather than ineligibility criteria.

Other eligibility criteria includes: [1] female sex; [2] age 18–45 years; [3] gestational age of > 4 weeks through 3 months postpartum; [4] receiving care from a participating practice, [5] able to communicate in written and spoken English; and [6] cognitively able to give informed verbal consent. If patients are not eligible during phone screening due to an EPDS< 10 but are otherwise interested in and eligible for participation, they are entered into a holding pool and re-contacted by phone at subsequent pregnancy and early postpartum time points. They are recruited into the study cohort at the time point they have onset of depressive symptoms (EPDS ≥10).

#### Patient participant-level assessments and study procedures

Patient participants recruited in phase 2, depending on their recruitment and first interview time point (4–24 weeks gestation, 32–40 weeks gestation, or 1–3 months postpartum), will be followed two to four more times until 13 months postpartum. As outlined in Table 3, several assessments are to be collected longitudinally. Patient participants consent to audiotaped interviews for the telephonic data collection. Total assessment time was pre-tested to assure calls did not exceed a time burden of greater than 60 min. Standardized or psychometrically validated tools were chosen to screen for other comorbid psychiatric illnesses, as opposed to a diagnostic interview, to limit respondent burden and maintain the practicality and feasibility that is imperative for this real-world study. In addition to the EPDS, MDQ, and 4Ps measured initially, post-traumatic stress disorder is screened for with the well-validated PTSD Screen-Civilian Version (PCL-C) [58], and generalized anxiety with the 7-item Generalized Anxiety Disorder Assessment (GAD-7) [59]. Mother infant bonding is assessed with the 10 item shortened version of the Postpartum Bonding Questionnaire (S-PBQ) [60]. Access to care is measured using the Barriers to Access to Care Evaluation scale (BACE), a 30-item tool that assesses barriers to accessing mental health care and includes a ‘treatment stigma’ subscale [61]. Patient participants’ beliefs and supports are assessed using the 30-item version of the Patient Attitudes towards and Ratings of Care for Depression (PARC-D), which allows the interviewer to explore the likelihood that a patient would use specific therapies for depression. A short, 14-question version of the Defense Style Questionnaire (DSQ-28) is used to screen for immature defenses which correlate with persistent depression. Finally, a structured interview is conducted to assess utilization of depression treatment, and barriers and facilitators to treatment participation. Other relevant survey questions (e.g. demographics) are collected at baseline, and obstetric and delivery outcomes at first postpartum visit (Table 3). Additionally, the cost and potential indicators of savings related to the MCPAP for Moms and PRISM interventions will be captured via measures and data sources as indicated in Table 4.

**Table 3:** Patient Participant-Level Measures

Outcome/ Endpoint	Measure	Administration	Time Points*				
			0–24 wks GA	32–40 wks GA	1–3 mos PP	5–7 mos PP	11–13 mos PP
Demographics	➢ Structured interview	Research Coordinator (RC) administered	√^†^	√^†^	√^†^		
Depression Severity	➢ EPDS	RC administered	√	√	√	√	√
Comorbid Anxiety Disorders	➢ GAD-7 ➢ PCL-C	RC administered	√	√	√	√	√
Knowledge, Attitudes, And Treatment Participation	➢ Structured interview assessing mental health treatment initiation and sustainment and barriers and facilitators to treatment participation	Structured interview with RC	√	√	√	√	√
Help-Seeking	➢ BACE ➢ PARC-D	RC administered	√	√	√	√	√
Infant Bonding	➢ S-PBQ	RC administered			√	√	√
Immature Defenses	➢ DSQ-28 (14 Questions)	RC administered			√		√
Obstetric Outcomes	➢ Structured interview assessing obstetric course, birth outcomes (e.g. birth weight, preterm delivery) and infant outcomes	Structured interview with RC			√	√	√

Table 3 represents the time points and frequency at which different patient participant measures are administered according to the PRogram In Support of Moms (PRISM) protocol for the Randomized Controlled Trial (RCT) phase of this study. Measures listed above do not include pre-screen and screening measures done before first time point, including baseline characteristics, MDQ (mood disorder questionnaire), and 4Ps (pregnancy, past, partner, parents) substance abuse screen. Patient participants that are recruited from the holding pool at later gestational ages or postpartum may not complete all measure time points. *Time Points refer to participant’s gestational age (GA) period or postpartum (PP) period in weeks (wks) or months (mos). ^†^ Demographic questions asked at the time of the initial study phone call with recruitment windows corresponding with the first half of pregnancy, second half of pregnancy, and early postpartum.

**Table 4:** Cost of PRISM and Indicators of Potential Savings

Outcome/ Endpoint	Measure	Data Source	Time frame
Cost of PRISM	➢ Startup costs ➢ Cost of implementation includes implementation team meetings and initial training (including the opportunity costs incurred by training participants) and other investments necessary for implementation	➢ Participation in, number and length of implementation meetings, trainings to prepare for PRISM implementation	Ongoing
	➢ Operational costs: PRISM general	➢ Participation in, number and length of PRISM sustainment meetings; cost of depression registry	Ongoing
	➢ Operational costs: Providers, staff, and navigator	➢ PRISM Navigator, provider, and staff time sampling	Occurs every 6 months for two years after intervention implementation
	➢ Operational costs: providers and staff	➢ PRISM provider and staff time sampling	Occurs every 6 months for two years after intervention implementation
Cost of MCPAP for Moms	➢ Operational costs: MCPAP for Moms (already in place)	➢ MCPAP for Moms data	Ongoing
Indicators of cost Saving Potential	➢ Compare hospitalization rates (medical, obstetric, and psychiatric) for perinatal patients receiving care from PRISM versus MCPAP for Moms	➢ Self-report during structured interviews with patients ➢ Cost per hospitalization based on average cost, obtained from Massachusetts Health Policy Commission, a publicly available de-identified database	Ongoing
	➢ Birth outcomes: birth weight, preterm delivery ➢ Infant outcomes: hospitalizations (NICU, special care nursery)	➢ Self-report during structured interview with patients	Ongoing

Table 4 represents the costs and indicators of potential savings measures for the PRogram In Support of Moms (PRISM) protocol versus the Massachusetts Child Psychiatry Access Program (MCPAP) for Moms. Time frames for data collection are varied.

Patient participant treatment initiation and sustainment will be operationalized as attendance at and number of mental health visit(s), receipt of prescription by an obstetrician, and adherence to medication regimen.

### Statistical analysis, power and sample size estimation for phase 2 RCT

Aligned with the overall study aims, there are two main hypotheses for which data analyses of the phase 2 RCT are planned. Hypothesis 1 (primary outcome) is that *perinatal patient participants with depressive symptomatology receiving care from practices enrolled in PRISM will experience more improvement in depression symptoms than patients receiving care from MCPAP for Moms only practices (2 point difference-of-difference in EPDS).* To specifically compare the difference in EPDS score over time between intervention arms, the treatment by time interaction will be evaluated. All analyses will be conducted using an intent-to-treat framework. Hypothesis 2 (secondary outcome) is that *perinatal patient participants with depressive symptomatology receiving care from practices enrolled in PRISM will have improved treatment initiation and sustainment as compared to patients receiving care from MCPAP for Moms only practices.* Patient participant treatment initiation and sustainment can be represented as dichotomous variables. Additionally this outcome can be assessed by counting the number of mental health visits within a prescribed timeframe. As in hypothesis 1 analyses, the treatment by time interaction will be of primary interest and adjustment for potential confounders (e.g., patient depressive symptom severity, non-white race/ethnicity, insurance status, and PRI).

Power and sample size estimates were based on hypothesis 1. Cluster randomized trials pose unique problems for sample size estimation and power. Within-cluster similarities require samples sizes to be larger than when using individual randomization [62]; thus an inflation factor representing the correction to the variance was used to calculate an appropriate sample size.

Based on a prior study involving perinatal participants with depressive symptoms, where the mean baseline EPDS score was 14.8 (SD = 5.3) and the mean final EPDS score was 9.7 (SD = 5.3) [37], we expect PRISM to cause a decrease in EPDS score of 5 points. We also expect the MCPAP for Moms only intervention to result in a decrease of 3 points in the EPDS resulting in a net difference between the two treatment arms of two points at follow-up [35]. Basing our sample size calculations on a two-sided, two-group, t-test of means with equal N’s and alpha = 0.05, we will have 80% power to detect this 2 point (small to medium Cohen effect size) difference at follow-up (effect size = 0.377) when the sample size is 112 patient participants per arm.

To account for clustering, we further increased these estimates by an inflation factor given by the equation: *IF = [1 + (m – 1) ρ*], where m represents the number of patients per practice, and *ρ* represents the intraclass correlation coefficient (ICC), the ratio of between-cluster variation to total variability. We estimate the ICC will be approximately 0.001 similar to values seen in other studies [63]. Calculating the inflation factor and adjusting previous power calculations, we seek to maintain 80% power to detect the desired difference, thus we will need a sample size of 115 patient participants per arm. Further, inflating our sample to account for 20% noncompliance and dropout reveals that we will require a final sample size of 150 patient participants per arm (5 practices per arm at 30 patient participants per practice). Of the 10,500 pregnant patients that will be available to be screened from participating practices, we conservatively expect that 10% (*n* = 1,500) [2, 64] will screen positive for depression on the EPDS (EPDS ≥10). Of those, we expect that 25% (*n* = 375) will not meet eligibility criteria for the study and that 50% (*n* = 750) will consent to the study. Thus, we plan to recruit three patient participitants per week over a period of two years to reach our sample size target of 300.

Based on our pilot work [35, 47] and our well-established approaches for recruiting participants and minimizing attrition/increasing retention, attrition rates of less than 15% among Ob/Gyn clinics/practices and less than 20% dropout of patient participants are expected. The run-in phase was conducted to ensure that the study procedures are feasible for the participating practices. The Ob/Gyn practices are regularly engaged by phone, email, and in person meetings. A retention rate of ≥80% of patient participants is anticipated because: [1] first contact is close to the initial assessment; [2] detailed locator information for the participant and two alternate contacts are obtained; [3] multi-method engagement strategies are used to proactively reach out to participants; [4] patient participant calls are scheduled at flexible times including evening availability; and, [5] patient participants are remunerated $25 for each completed assessment and then $30 at the final completed assessment.

Regardless of whether a participant has a complete record at each assessment, all available data will be used. Completely at random, missing at random, or missing not at random/non-ignorable [65, 66] data will be assessed and will be addressed appropriately.

### Quality assurance, subject protections and data requests

Standard operating procedures for quality assurance/quality control (QA/QC) take place in the UMass Medical School Quantitative Methods Core. Staff are trained and provided clear guidance on data collection, quality assurance, and standardized procedures for transmitting data in accordance with protocols. Our data collection software systems are programmed with validation rules. Study staff administer assessments using electronic data capture with software systems that contain built-in checks for validity, consistency, logic, appropriate skip patterns and normal range values.

The PI, Co-PIs and Project Director provide oversight and direction to the study staff to ensure study protocol and procedures are followed and any issues are tracked and corrected. The Project Director trains all study staff to conduct interviews using a standardized and neutral assessment technique. The Project Director reviews a sampling of recorded patient consents and assessments for the first month that study staff conduct assessments independently. A 10% random sample of the assessments are reviewed annually to ensure that the data entered is consistent with participants’ answers during audiotaped assessments and for consistency of interview technique across study staff. The Project Director then provides feedback to study staff regarding interview approach and errors in data entry, and develops a plan to avoid any further errors.

Any protocol deviations are reviewed by the PI who determines if modifications to the study protocol or consent form are required. A Data Safety Monitoring Board (DSMB) was convened and acts in an expert, independent advisory capacity. Protocol deviations are reported to the DSMB as indicated. The DSMB includes three members with expertise in perinatal psychiatry, Ob/Gyn, and methodology. The DSMB responsibilities are to: [1] conduct interim and final evaluations of the study, including analysis of aggregate and individual participant data related to safety, data integrity and the overall conduct of the study; [2] consider factors external to the study when relevant information becomes available, such as scientific or therapeutic developments that may have an impact on participant safety, scientific integrity, or the ethics of conducting the study; [3] protect the safety of study participants; [4] review and evaluate ad hoc safety issues concerning the study at the request of the investigator(s); and, [5] make recommendations to the investigator(s) concerning continuation, termination, or other modifications of the study based on the observed benefits or adverse effects of the study. The DSMB meets every six months. Additionally it meets, 15 months after the start of the RCT data collection, upon completion or termination of the study, within 48 h of an adverse event or protocol violation, and on an ad hoc basis if there is deemed to be an imminent participant safety issue. At each meeting, the DSMB determines whether the study should continue as planned, proceed with modifications, or be terminated. Justification to terminate may be due to the DSMB’s analysis that there is an overwhelming safety issue or that recruitment is insufficient. The complete DSMB charter is available upon request.

The study protocol is in compliance with the NIH Data Sharing Policy and Implementation Guidance (http://grants.nih.gov/grants/policy/data_sharing/data_sharing_guidance.htm) and the NIH Statements on Sharing Research Data and on Availability of Research Results: Publication, Intellectual Property Rights, and Sharing Research Resources. Data will be categorized to meet requirements for protected health information, stripped of all study identification codes, and will be transmitted in encrypted or otherwise secure files for use according to the plans and policies of NIH, CDC and participating investigators and institutions. In accordance with the PRISM Publication Guidelines and Agreement (available upon request), data requests are submitted to the publication committee and minimally include the primary question, proposed analyses/sample, and purpose. The PRISM publication committee includes UMass and CDC team members. Prior to receiving PRISM data, all researchers are expected to sign a data use agreement.

## Discussion

Recognizing maternal mental health as critical to quality perinatal care and maternal safety, the Council on Patient Safety in Women’s Health Care established a maternal mental health safety bundle summarizing existing evidence informed recommendations, paired with resources [15, 67]. However, unlike prior bundles including those addressing obstetric hemorrhage [68] and hypertensive crisis [69], the maternal mental health bundle presents unique implementation challenges as its scope spans across multiple disciplines (e.g. obstetrics, psychiatry, psychology and others) and is beyond the hospital maternity unit, precluding it from being adequately addressed with usual approaches like standardized order sets, drills, and dedicated carts [68, 69]. Thus, interventions are needed that address patient, provider, and system level barriers to the successful integration of depression care into outpatient obstetric practice [20–22].

Despite the clear need, there are a limited number of interventions aimed at integrating obstetric and depression care [14, 34]. Existing interventions that demonstrate feasibility, effectiveness, and acceptability [34] are rigid in their structure, and, with limited exceptions [35–39], have focused almost exclusively on process rather than outcome measures, or have either not addressed sustainability or do not exist beyond their grant funding and research team support [34]. In response, in this cluster-RCT, we are rigorously comparing the extent to which PRISM improves depression symptomatology and treatment participation among perinatal women as compared to MCPAP for Moms while also estimating costs and indicators of potential savings.

Our design allows us to evaluate two interventions, MCPAP for Moms and PRISM, which target all obstetric practices in the state and individual practices respectively. Both MCPAP for Moms and PRISM recognize that obstetric practices are complex evolving systems and thus predetermined, one-size fits all approaches are limited in their effectiveness. This cluster-RCT recognizes the practice as the natural unit of randomization. Moreover, it innovates and advances the field by integrating obstetric and psychiatric care by invoking implementation research principles and participatory action research in a randomized controlled trial [70]. Important concepts of participatory action research include that key intervention elements are locally implemented based on collaborative discussions, that on-site champions are collaborators and share authority with study team, and that local insights inform implementation processes and general understanding to improve addressed issues [70].

The participatory action research framework allows practice-specific goals and processes to vary within boundaries facilitated by a standardized menu of options, that are offered to practices and customized to meet their needs. This allows for the rigor of RCTs to be generalizable in the context of organizations which all have unique characteristics and conditions. Accordingly, PRISM is a multi-pronged intervention with a structured implementation process that empowers and supports practices in identifying practice-specific goals and solutions, through an iterative approach that maintains core components across all practices, while allowing for customization to specific practice environments. PRISM is also unique in that it builds on MCPAP for Moms by adding more intensive and proactive practice-specific goals and activities. MCPAP for Moms is a statewide program that leverages limited resources to improve access to mental health resources across the state of Massachusetts including ready access to perinatal psychiatrists [40, 42]. In the first 5 years since the program went live, MCPAP for Moms is seen as a national model and has influenced state and national policies [41, 71–73]. MCPAP for Moms was the inspiration behind the *Bringing Postpartum Depression out of the Shadows Act* (H.R. 3235) [71, 74], which was consolidated into *twenty-first Century Cures* and signed into law. This legislation led to the appropriation of $5,000,000 in the FY 18–19 federal budget for the United States Health Resources and Services Administration (HRSA) to administer grants for other states to establish perinatal psychiatry access programs, similar to MCPAP for Moms [75, 76]. Seven U.S. states (FL, KS, LA, MT, NC, RI, VT) were recently awarded the grants each to establish their own perinatal psychiatry access programs [77].

Compared to MCPAP for Moms, PRISM is more focused on the individual practice level, and uses existing infrastructure and resources within practice settings [35, 47]. Our study carries the potential for its results to have policy and public health implications because both interventions have the potential to be feasible, sustainable, and transportable to other practice settings. Given this potential, indicators of potential cost-savings will be collected in phase 2 as successful dissemination of any program is reliant not only on positive results but also on financial sustainability and demonstration of short- and/or longer-term investment return with consideration of programmatic costs, and effect on maternal and child outcomes.

Another unique feature of our study design is that it allows us to recruit women at three time points in order to account for the different time periods for depressive symptom onset. Our holding pool will also allow us to follow a cohort of women and monitor for the onset of depression and factors associated with symptom onset.

The run-in phase of this protocol demonstrated the feasibility of practice and patient participant recruitment. Our randomization approach protects against heterogeneity within practices as well as imbalances at the individual level caused by practices having different patient profiles.

Effective investigations of programs that benefit perinatal women by addressing their mental health are greatly needed. The public health impact of such interventions has great potential given the intergenerational effects of perinatal depression. Our MCPAP for Moms and PRISM interventions target obstetric practices with the goal of increasing their capacity to adequately address depression in perinatal women. They each capitalize on and extend beta-testing [47], pilot trials [35], and state-wide program development [40, 42], all of which have identified continued gaps in the perinatal depression care pathway and have informed iterative changes with the goal of continued improvements for the benefits of mothers and their mental health. Once the full trial is complete, we will disseminate findings via a multi-dimensional strategy that will involve traditional academic and scientific venues, in addition to advanced social media applications, and other contemporary approaches.

## Additional files

Additional file 1: The study timeline includes major study milestones and corresponding dates. (PDF 130 kb)

Additional file 2: There are 10 practices that were randomized for this study. The table includes the names of the practices, the region of Massachusetts in which they are located, and their location (city or town). (DOCX 13 kb)

## Data Availability

Not applicable as this is a study protocol manuscript and does not include analyses of any data.

## Abbreviations

4Ps: 4 P’s (*Pregnancy, Past, Partner, Parents)* substance abuse screen
BACE: Barriers to Access Care Evaluation
DSMB: DataSafety Monitoring Board
DSQ-28: Defense Style Questionnaire
EPDS: Edinburgh Postnatal Depression Scale
GAD-7: Generalized Anxiety Disorder 7-item scale
HIPAA: Health Insurance Portability and Accountability Act
ICC: Intraclass Correlation Coefficient
MCPAP for Moms: Massachusetts Child Psychiatry Access Program for Moms
MDQ: Mood Disorder Questionnaire
NICU: Neonatal Intensive Care Unit
Ob/Gyn: Obstetrics and Gynecology
PARC-D: Patient Attitudes toward and Ratings of Care for Depression
PCL-C: PTSD CheckList – Civilian version
PREPD: Practice Readiness to Evaluate and address Perinatal Depression scale
PRI: Practice Readiness Index
PRISM: PRogram In Support of Moms
QA/QC: Quality Assurance/Quality Control
RC: Research Coordinator
RCT: Randomized Controlled Trial
S-KAP: Smoking Knowledge, Attitudes, and Practice instrument
S-PBQ: Short Postpartum Bonding Questionnaire
