# Supplementary figures and images for: The PRogram In Support of Moms (PRISM): study protocol for a cluster randomized controlled trial of two active interventions addressing perinatal depression in obstetric settings
Source: BMC Pregnancy Childbirth. 2019 Jul 22;19:256. doi: 10.1186/s12884-019-2387-3 (PMC6647165; doi:10.1186/s12884-019-2387-3)

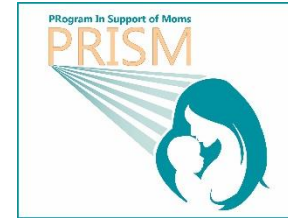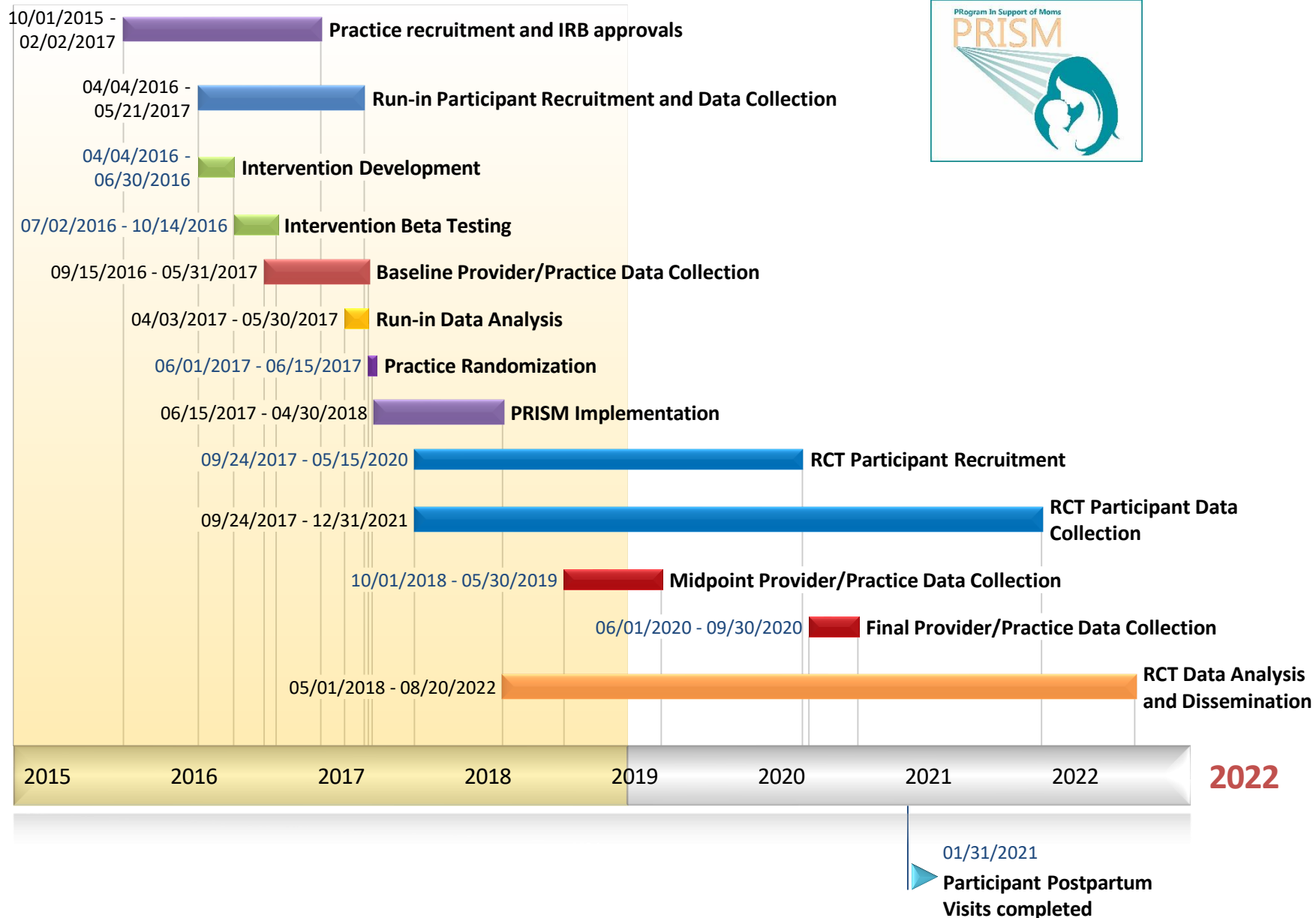

Supplement: Supplementary file 1 — The study timeline includes major study milestones and corresponding dates. (PDF 130 kb) [file 12884_2019_2387_MOESM1_ESM.pdf]
