# Supplementary material for: The PRogram In Support of Moms (PRISM): study protocol for a cluster randomized controlled trial of two active interventions addressing perinatal depression in obstetric settings
Source: BMC Pregnancy Childbirth. 2019 Jul 22;19:256. doi: 10.1186/s12884-019-2387-3 (PMC6647165; doi:10.1186/s12884-019-2387-3)
Supplement: Supplementary file 2 — There are 10 practices that were randomized for this study. The table includes the names of the practices, the region of Massachusetts in which they are located, and their location (city or town). (DOCX 13 kb) [file 12884_2019_2387_MOESM2_ESM.docx]

**Additional File 2 – 10 Participating practices in PRISM study**

| **Practice name** | **Practice Region** | **Practice location** |
| --- | --- | --- |
| UMass Memorial Medical Center – Community Women’s Care | Central MA | Worcester, MA |
| Montachusett Women's Health | Central MA | Leominster, MA |
| Cape Cod Hospital Obstetrics & Gynecology Clinic | Eastern MA | Hyannis, MA |
| Reliant Medical Group Obstetrics & Gynecology | Central MA | Worcester, MA |
| Gyn-OB Associates of Fall River – Southcoast Health | Eastern MA | Fall River, MA |
| Southcoast Physicians Group | Eastern MA | New Bedford, MA |
| Baystate Wesson Women’s Clinic | Western MA | Springfield, MA |
| Pioneer Women’s Health | Western MA | Greenfield, MA |
| Beth Israel Deaconess Medical Center | Eastern MA | Boston, MA |
| Tufts Women’s Care | Eastern MA | Boston, MA |
